# Use of probiotics to reduce infections and death and prevent colonization with extended-spectrum beta-lactamase (ESBL)-producing bacteria among newborn infants in Tanzania (ProRIDE Trial): study protocol for a randomized controlled clinical trial

**DOI:** 10.1186/s13063-021-05251-3

**Published:** 2021-04-29

**Authors:** Kanika Kuwelker, Nina Langeland, Iren Høyland Löhr, Joshua Gidion, Joel Manyahi, Sabrina John Moyo, Bjørn Blomberg, Claus Klingenberg

**Affiliations:** 1 Norwegian National Advisory Unit on Tropical Infectious Diseases, Haukeland University Hospital, Haukelandsbakken, 5009 Bergen, Norway; 2 Department of Clinical Science, University of Bergen, Laboratory Building, Haukeland University Hospital, Jonas Lies veg 87, 5021 Bergen, Norway; 3 Department of Medical Microbiology, Stavanger University Hospital, Gerd Ragna Bloch Thorsens gate, 4011 Stavanger, Norway; 4 Department of Paediatrics, Haydom Lutheran Hospital, Mbulu, Manyara Tanzania; 5 Department of Microbiology and Immunology, Muhimbili University of Health and Allied Sciences, MUHAS, P.O. Box 65005, Dar es Salaam, Tanzania; 6 Department of Paediatrics and Adolescence Medicine, University Hospital of North Norway, Tromsø, Norway; 7 Paediatric Research Group, Faculty of Health Sciences, University of Tromsø-Arctic University of Norway, Tromsø, Norway

**Keywords:** Newborn, Infants, Probiotics, Gut colonization, Extended-spectrum beta-lactamase, ESBL, Enterobacteriaceae, Infant mortality, Hospitalization, Microbiota, Resistome, Randomized controlled trial

## Abstract

**Background:**

Extended-spectrum beta-lactamase-producing *Enterobacteriaceae* (ESBL-E) has emerged as an urgent global health threat and is by the World Health Organization ranked as priority 1 among pathogens in need of new treatment. Studies have shown high mortality in Tanzanian children with ESBL-E infections. Gut colonization of ESBL-E, which is a potential risk factor of ESBL-E infections, is reported to be very high among children in Tanzania. Probiotics may potentially reduce gut colonization of multidrug-resistant bacteria. However, there is limited data on whether probiotics may reduce ESBL-E carriage in infants. The ProRIDE Trial aims to evaluate whether the use of probiotics can reduce morbidity and mortality among infants in Haydom, Tanzania, and whether this effect is associated with a reduction in ESBL-E colonization and/or infections.

**Methods/design:**

This large randomized double-blinded placebo-controlled trial aims to recruit 2000 newborn infants at Haydom Lutheran Hospital and the surrounding area in the period of November 2020 to November 2021. Participants will be enrolled from days 0 to 3 after birth and randomized to receive probiotics or placebo for 4 weeks. Participants will be followed-up for 6 months, during which three visits will be made to collect clinical and demographic information, as well as rectal swabs and fecal samples which will be subjected to laboratory analysis. The primary composite outcome is the prevalence of death and/or hospitalization at 6 months of age.

**Discussion:**

As the use of probiotics may give a more favorable gut composition, and thereby improve health and reduce morbidity and mortality, the results may have implications for future therapy guidelines in Africa and internationally.

**Trial registration:**

ClinicalTrials.gov NCT04172012. Registered on November 21, 2019

**Supplementary Information:**

The online version contains supplementary material available at 10.1186/s13063-021-05251-3.

## Introduction

### Background and rationale {6a}

Infections continue to be a considerable cause of death and disease among infants in low- and middle-income countries. In sub-Saharan Africa, infections contribute to three–forth of under-five mortality [1]. In Tanzania, the under-five mortality was reported at 55 per 1000 live births in 2016, whereof 42 deaths occurred before the age of one year [2]. An increasing prevalence of antimicrobial resistance (AMR) is among the most urgent global threats [3]. Previous studies of hospitalized children in Tanzania have shown that blood-stream infections (BSIs) caused by extended-spectrum beta-lactamase-producing Enterobacteriaceae (ESBL-E) caused a mortality rate of more than 70%, compared to 20% or less for malaria infections and BSI with susceptible bacteria [4, 5]. A potential risk factor of ESBL-E infection is gut colonization with ESBL-E [6–8]. Colonization rates have now evolved towards a global pandemic with developing countries being affected most [9]. Our research group has previously shown that around two–third of Tanzanian infants below three months of age had fecal carriage of ESBL-E [10].

Probiotics are live microorganisms that, when administered in adequate amounts, confer a benefit to the host. Probiotics are currently widely used in newborns and children [11]. They have proven beneficial effects in preterm infants reducing necrotizing enterocolitis and sepsis [12, 13], and this has been observed both in high-, middle-, and low-income countries [14]. In term infants, a study from India was conducted where more than 4500 term-born infants were randomized to receive either the probiotic bacteria *Lactobacillus plantarum* along with fructo-oligosaccharide, a plant-derived prebiotic (the combination of probiotic and prebiotic is coined “synbiotic”) or placebo [15]. This simple intervention reduced the composite outcome of severe infections and/or death by 40%, from 9.0% in the placebo group to 5.4% in the synbiotic group.

A stable and resilient commensal gut microbiota is essential for “colonization resistance”; the ability to prevent intestinal colonization and invasion by pathogens. Resurrecting the gut microbiota by the use of probiotics has been put forward as a new strategy in combatting intestinal carriage of AMR-bacteria [16]. To what extent probiotics can reduce the spread of AMR is still under investigation [17], but probiotic bacteria produce bacteriocins that improve mucosal integrity and thereby may reduce the pathogenic bacterial population and promote “colonization resistance” [16]. There is limited data on which probiotic strains that most effectively may reduce AMR in children. In adults and older children, lactobacilli are often used for conditions like antibiotic-associated diarrhea and gastroenteritis [18, 19]. In breastfed infants, bifidobacteria constitute more than 80% of the gut microbiota, and when given in combination with human milk, it has decreased the share of *Enterobacteriales* in the feces of infants [20]. Breastfeeding, which promotes bifidobacterial dominance, appears to protect infants from colonization with AMR bacteria [21, 22]. Probiotic bifidobacterial strains were also shown to inhibit the transfer of beta-lactam resistance among Enterobacteriaceae in gnotobiotic mice [23] and reduce AMR gene carriage in a randomized trial of 60 term infants [24]. In our own studies among extremely preterm infants supplemented with a probiotic product containing bifidobacteria and lactobacilli, we found no ESBL-E in stool samples despite massive exposure to antibiotic therapy. In contrast, ESBL-E was detected in the stool of moderate preterm infants and full-term infants not given probiotics and with less or no antibiotic exposure [25]. This indicates that a probiotic combination may prevent AMR development, but larger studies are needed to confirm these promising results.

Except from the study by Panigrahi et al. [15], no other large randomized clinical trials (RCT) have investigated whether bifidobacterial probiotic supplementation can reduce mortality and infection-related morbidity in infants in low-income countries. More specifically, no large RCTs have investigated whether probiotics may reduce ESBL-E colonization in infants.

We plan to conduct a large RCT among term infants in North-East Tanzania to evaluate whether the use of probiotics can reduce infection-related morbidity and mortality, similar to the Panigrahi trial [15]. Secondly, we will investigate whether potentially beneficial effects are associated with a reduction in ESBL-E colonization and/or infections.

### Objectives {7}

The research question is Among term-born healthy infants in Tanzania (P-population), will treatment with probiotics (I-intervention) compared to placebo (C-comparison) lead to a 40% reduction in hospitalizations and death, and in reduced fecal colonization of ESBL-E (O-outcome)?

### Overall objective of the study

The present randomized placebo-controlled clinical trial will evaluate whether administration of probiotics for the first 4 weeks of life, compared to placebo, can reduce morbidity and mortality by preventing ESBL-E carriage and/or infections among infants in Haydom and surrounding area, Tanzania at follow-up 6 weeks and 6 months after birth.

### Specific objectives of the study

1. To assess and compare mortality among groups receiving probiotics or placebo.
2. To determine and compare episodes of infections (i.e., sepsis, diarrhea) leading to hospitalization among groups receiving probiotics or placebo.
3. To determine and compare the prevalence of ESBL-E colonization among groups receiving probiotics or placebo.
4. To determine and compare the bacterial causes of sepsis, including antimicrobial susceptibility patterns, among groups receiving probiotics or placebo.
5. To assess and compare the effect of probiotics on growth among groups receiving probiotics or placebo.
6. To determine and compare the gut microbiota composition and diversity among groups receiving probiotics or placebo.
7. To determine and compare the gut metabolome composition among groups receiving probiotics or placebo.
8. To determine and compare the gut inflammatory markers among groups receiving probiotics or placebo.
9. To determine the bacterial causes of sepsis, including antimicrobial susceptibility patterns for all children under 1 year of age admitted with fever during the study period.

### Trial design {8}

The ProRIDE Trial is a double-blinded, placebo-controlled RCT with two arms. More specifically, it is a parallel group, superiority study with 1:1 allocation of the randomized product (active probiotics or placebo).

## Methods: participants, interventions, and outcomes

### Study setting {9}

Patients will be recruited among newborns; born at Haydom Lutheran Hospital (HLH) in North East Tanzania and out-born newborns in the surrounding area. HLH is located in the Mbulu district, at the western end of the Manyara Region in the North-Central Tanzania, about 300 km south-west from regional center Arusha. The HLH catchment area consists of four administrative divisions, three districts, and two regions. These are the Dongobesh and Haydom divisions in Mbulu District, the Basotu Division in Hanang District (Manyara Region), and the Nduguti Division in Iramba District (Singida Region). The hospital serves a population of more than two million people from five regions. Annually, there are more than around 3900 deliveries in the hospital, and in 2018, the neonatal mortality rate in Tanzania was around 21/1000 live births [26].

### Eligibility criteria {10}

#### Inclusion criteria

Healthy newborn infants with a birth weight (BW) equal or above 2.0 kg are eligible for inclusion. Newborn infants have to come from families who are long-term or permanent residents in the defined catchment area for this trial (30 km radius from HLH) in Tanzania. Parents have to be able and willing to complete study visit (including required study procedures) schedules over the 6 months proposed follow-up. This also includes that they, if possible, bring their child to the hospital in case of any intercurrent illness. Parents have to sign an informed consent form (ICF) and have to agree that the child cannot participate in another clinical trial during the study period.

#### Exclusion criteria

BW below 2.0 kg, refusal of informed consent and/or other health problems/illness including obvious congenital malformations.

### Who will take informed consent? {26a}

Pregnant women in their last trimester, visited at home or attending the last antenatal care visit at HLH, will receive oral and written information about all aspects of the study before giving consent to let their future child participate in the study. Trained research assistants who speak the local language will be in charge of obtaining informed consent before recruitment.

### Additional consent provisions for collection and use of participant data and biological specimens {26b}

On the consent form, participants will be informed about the collection of stool samples and clinical data and that participating in the study is completely voluntary and that parents have the right to withdraw the inclusion of their child at any time.

## Interventions

### Explanation for the choice of comparators {6b}

Currently, there is no standard therapy for preventing ESBL-E colonization in term infants and thus, “the-best-available therapy” is no therapy. Therefore, the comparator group will receive an inactive placebo agent.

### Intervention description {11a}

The active group will receive the investigational product (IP); a commercially available multi-strain probiotic product (LaBiNIC® probiotic drops, Biofloratech Ltd., Surrey, UK). The product is already widely used in the UK (at hospitals in the National Health Service) and in other European countries [27, 28]. Previous studies in developing countries have used from 7 days (India [15]) to 60 days (Botswana [29]) intervention with probiotic supplementation in infants.

Five drops (0.2 ml) of the IP contain two billion (total) of *Lactobacillus acidophilus NCFM*, *Bifidobacterium bifidum Bb-06*, and *Bifidobacterium infantis Bi-26* probiotics (equal amount of all three strains), suspended in pharmaceutical-grade capric MCT (medium-chain triglyceride) oil and Aerosil® 200 Pharma (inactive ingredients). Each bottle contains 5–5.5 ml and will be administered orally once daily for 4 weeks to “bottle empty” (five drops per day). The placebo group will receive placebo drops containing only the inactive ingredients; a capric MCT-oil and AEROSIL® 200 Pharma. AEROSIL® 200 Pharma is an additive for food and pharmaceutical products with a high-quality standard and with production processes based on the quality concepts such as ISO 9001, HACCP, FAMI-QS, and IPEC-GMP. The MCT-oil and AEROSIL® 200 Pharma are also constituents of the active IP. The placebo is produced by the same company that manufactures the IP (Biofloratech Ltd., Surrey, UK). The MCT-oil is used as a vehicle for the probiotic bacteria in the IP, and the taste and color are identical, and it will be produced and dispensed in identical bottles to the IP.

### Criteria for discontinuing or modifying allocated interventions {11b}

Study participants will be discontinued from participation in the study if any clinical adverse event (AE), laboratory abnormality, or other medical condition or situation occurs during the 4-week intervention. Study participants will also be discontinued from participation in a situation where continued participation is not in the best interest of the participants. Participants are free to withdraw from participating in the study at any time upon request. In any case, participants will be given appropriate care under medical supervision until the symptoms of any AE resolve or the participant’s condition becomes stable.

### Strategies to improve adherence to interventions {11c}

During recruitment, allocation and the first follow-up mothers/caretakers will receive information on how to administer the drops appropriately and the importance of completing the course. Adherence will also be evaluated during the first and second follow-up visits by observing the content of the bottles. Appropriate delivery of the IP cannot be verified by laboratory tests during the intervention.

### Relevant concomitant care permitted or prohibited during the trial {11d}

Even though the study products are not considered to be medicinal drugs, and studies have so far not revealed any serious adverse effects, study participants will be insured by the Strategis Insurance in Tanzania (https://www.strategis.co.tz/) for serious adverse events, including hospitalization, disability, and death.

All study participants, when they fall sick during the study period, will be treated according to the existing standard of care at HLH. In addition, the study will provide C-reactive protein analysis, blood culture bottles, and antimicrobial susceptibility testing of bacteria recovered from blood culture for all children under one year of age suspected of having sepsis. Malaria rapid test will be performed if clinically indicated (Care Start, USA). Laboratory results will be given to the attending physician to assist the patient management. During recruitment, parents will have to agree for the child not to participate in another study during the study period.

### Provision for post-trial care {30}

As there is no anticipated harm for trial participation, we have not planned for compensation or post-trial care.

### Outcomes {12}

#### Primary outcome

Prevalence (%) of the composite outcome death and/or hospitalizations at 6 months of age.

#### Secondary outcomes

- Rates (%) of ESBL-E colonization at 6 weeks and 6 months of age.
- Rates (%) of hospitalizations up to 6 months of age.
- Rates (%) of confirmed sepsis (blood culture-confirmed) episodes up to 6 months of age.
- Growth monitored by length and weight up to 6 months of age.
- Stool microbiota composition including resistome analysis (metagenome sequencing) at 6 weeks and 6 months of age.
- Stool metabolome composition at 6 weeks and 6 months of age.
- Stool inflammatory markers at 6 months of age
- Genetic characteristics of ESBL-E from colonization and clinical samples (targeted screening).

### Participant timeline {13}

See “Fig. 1”.

**Fig. 1:**
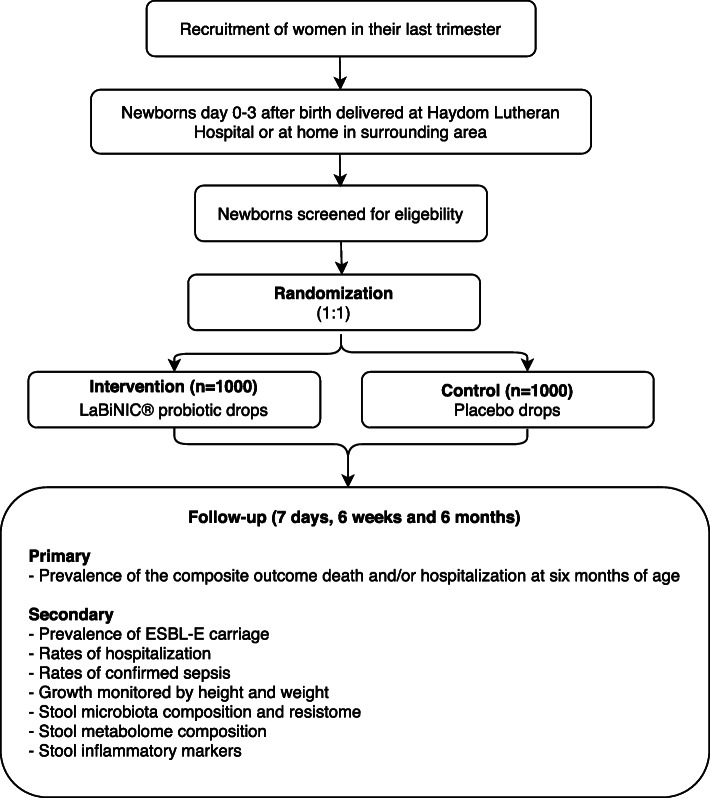
Trial flow chart. Abbreviations: ESBL-E extended-spectrum beta-lactamase Enterobacteriaceae; MDR multidrug resistant

### Sample size {14}

The infant mortality rate in Tanzania was in 2018 reported at 3.8% [26]. We do not have exact data for the Mbulu district, but we assume a similar rate of approximately 4%. Serious infections leading to hospitalization are probably equally common, but hospitalization rates may depend on available access. In the Panigrahi study [15], the synbiotic product was given for only 7 days, and it reduced the composite outcome of death and/or infection from 9.0% to 5.4% during the first 2 months of life. In our study, the intervention will continue for around 4 weeks and follow-up will continue until 6 months of life. We consider a similar reduction in the composite outcome of death and infections leading to hospitalizations from 9.0% to 5.4%, as in the Panigrahi study [15], to be clinically relevant. To find this difference with a study power of 85% at 5% significance level, we will need a sample size of minimum of 924 cases in each arm, thus 1848 infants. In order to allow for drop-out, we aim to include 2000 infants. We believe it is realistic to include this number of infants within a 12-month recruitment period. With this number of infants, we will also have sufficient power to detect a minimum of 30% reduction in ESBL-E colonization rate (from around 50% to 35%) at 6 months of age.

**Table 1:** Trial schedule procedures

Study period					
	Enrolment	Allocation	Post-allocation		
Timepoint	Screening	Baseline	7 days^*^	6 weeks	6 months
**Enrolment**					
Informed consent mother/caretaker	x				
Eligibility screening newborn		x			
Randomization newborn		x			
**Interventions**					
Probiotics		x	x		
Placebo		x	x		
**Assessment**					
Rates of confirmed sepsis episodes				x	x
Rates of hospitalization				x	x
Growth by height and weight				x	x
Stool microbiota composition and resistome				x	x
Stool metabolome composition				x	x
Stool inflammatory markers					x
Genetic characteristics of ESBL-E and other MDR isolates				x	x
Prevalence ESBL-E carriage				x	x

Abbreviations: *ESBL-E* extended-spectrum beta-lactamase Enterobacteriaceae, *MDR* multidrug resistant

^*^Observe for adherence and discuss issues with care takers

**Table 2:** Monitoring plan

	1st quarter	2nd quarter	4th quarter	8th quarter
**Enrolment visit**	x			
**Follow-up visit**		x	x	
**Close up**				x

**Table 3:** Key activities of the clinical monitor

Site visits	When	Key activities
**Monitoring visit during enrolment**	One visit, 4 weeks since the start of enrolment.	- Inspection of all ICH GCP certificates in place - Verification of informed consent forms - Eligibility check - eCRF completion - Equipment, consumables, study material - Review laboratory sample management
**Monitoring visit follow-up—first**	One visit 6 months since the start of enrolment.	- Specimen collection procedure - Eligibility check - eCRF completion - Eligibility of source documents - Source data verification - Review AE/SAE reporting
**Monitoring visit follow-up—second**	One visit 12 months since the start of enrolment.	- Specimen collection procedure - Eligibility check - eCRF completion - Eligibility of source documents - Source data verification - Review AE/SAE reporting
**Close-up visit monitoring**	One visit 18 months since the start of enrolment (end of study)	- Last query resolution - Storage and archiving of documentation - Review, update, and finalization of IF

Abbreviations: *AE/SAE* adverse events/serious adverse events, *eCRF* electronic case report forms, *ICH GCP* International Conference on Harmonization Good Clinical Practice, *IF* investigator file

### Recruitment {15}

Pregnant women in their last trimester, visited at home or attending their last visit before delivery at HLH, will receive oral and written information about the study and asked for consent to let their future child participate in the study. A study research assistant speaking the native language will inform the woman about all aspects of the study, including the importance of performing such a study, and all procedures that will take place during the study period. A thorough explanation will also be given on how the IP/placebo will be administered orally once daily with five drops until the bottle is empty (4 weeks). Finally, the importance of completing the dosage of the IP/placebo for the 4-week intervention period will also be explained to the woman. Information will be given orally and by information included in a written informed consent form (ICF).

If the woman, after this information, agrees for her child to participate in the ProRIDE Trial, she will be asked to give written consent to screen and enroll the newborn baby in the study. The woman will receive a special information card developed for the ProRIDE Trial, after delivery and inclusion. During and after delivery, the attending doctor/midwives will identify mothers who have consented to participate and inform the research assistant, who will help coordinate the study. After delivery, the newborn baby will be screened for eligibility into the study, and if the baby fulfills the inclusion criteria of the study, the baby will be enrolled and allocated to the randomized product (active probiotics or placebo), which will be initiated on days 0–1 for hospital deliveries or days 0–3 for home deliveries. The mother will again be explained and demonstrated how to administer the study product.

## Assignment of interventions: allocation

### Sequence generation {16a}

Prior to the start of the study, an independent researcher/statistician at the University of Bergen will be in charge of creating a computer-generated randomization list stratified by site of delivery (home/hospital) with the study identification number of patients, from 1 to 2000, and allocated investigational product or placebo. We have not planned for any restrictions to reduce the predictability of the randomization list.

### Concealment mechanism {16b}

Upon screening, the newborn infants will consecutively be allocated (randomized) to the next study identification number and given the corresponding investigational product/placebo, from the central storage facility at HLH. The IP and placebo are identical in taste and color and produced in identical bottles. A local pharmacist, who is not part of the study, will be provided with the randomization list and will also label the identical bottles, numbered from 1 to 2000.

### Implementation {16c}

A staff member at the University of Bergen who will not be involved in the trial will generate the allocation sequence using a computer software program. Research assistants at HLH, who will not have access to the randomization list, will be in charge of screening, recruiting, and assigning interventions to participants.

## Assignment of interventions: blinding

### Who will be blinded {17a}

Both care providers (study participants are infants) and investigators will be blinded for the assignment of the intervention.

### Procedure for unblinding if needed {17b}

A local pharmacist not involved in the study will have access to the assignment list. Therefore, the study principal investigator may request unblinding for treatment assignment if needed for medical care of a patient in an emergency situation. However, this is a very unlikely situation given the well-known safety of probiotics in infants, and the lack of known interaction with other important drugs used for any other required therapy.

Study participants will be discontinued from participation in the study if (i) any clinical AE during the 4-week intervention, laboratory abnormality, or other medical condition or situation occurs such that continued participation is not in the best interest of the participants, (ii) participants wish voluntary withdrawal, (iii) participants are free to withdraw from participating in the study at any time upon request and/or [4] in any case participants will be given appropriate care under medical supervision until the symptoms of any AE resolve or the participant’s condition becomes stable.

## Data collection and management

### Plans for assessment and collection of outcomes {18a}

#### Data collection

The information will be recorded on electronic CRFs (eCRFs) using REDCap® (Research Electronic Data Capture). For enrolled infants, four different case reports forms (CRFs) will be used to collect clinical and demographic information—three for the study visits and one for AE/unscheduled visits due to hospitalization/outpatient clinic attendance for participants who fall sick during the study period. The initial data at enrolment will also be filled with the mother’s demographic and clinical information (Table 1).

All staff members involved in data collection will receive training in utilizing the REDCap tool, as well as training in Good Clinical Practice (GCP) guidelines. Quality assessment of the data will be done by the internal clinical staff and consent forms and eCRFs will be checked by external monitors.

#### Laboratory methods

Fecal samples and rectal swabs collected at 6 weeks and 6 months will be transported to Norway for the following analyses (Table 1):

- Rectal swab samples (*n* = 4000) will be screened for ESBL-E using selective ESBL-screening agar (ChromID ESBL and ChromID Carba, BioMerieux). All samples will be plated on non-selective plates for growth control. ESBL-E isolated will be identified using MALDI-TOF and subsequently frozen for genetic analysis (whole genome sequencing and plasmid analysis).
- Fecal samples (*n* = 4000) will be subjected to gut microbiota and resistome analysis using a metagenomics approach. This will be done on DNA extracted from the fecal samples which have been collected and stored using a purpose-designed sampling kit (OMNIgen GUT kit, DNA Genotek, Ottawa, Canada). These kits have been evaluated using fresh stool samples compared to samples stored in an OMNIgen GUT sampling tube. There was no change in gut microbiota profile after the incubation at 50 °C for 3 days consecutively and following six freeze-thaw cycles (information from the manufacturer).
- Fecal samples (a subset of *n* = 500 samples) will be subjected to gut metabolome studies. We will use purpose-designed commercially available sampling kits (OMNIgen GUT kit, DNA Genotek, Ottawa, Canada) with proven stability of metabolites under storage at ambient temperatures for up to 14 days.

### Plans to promote participant retention and complete follow-up {18b}

Firstly, all pregnant women will receive sufficient information on what is expected from them if they allow for their newborn to be recruited and the importance of follow-up. During the study, we have scheduled three follow-up visits where assigned research assistants will visit households of participants; thus, participants will not be burdened with the additional inconvenience of traveling to the hospital and potentially falling out of the study. Furthermore, all enrolled newborns will receive insurance that will compensate for expenses related to hospitalization/hospital visits during the trial period. Mothers/caretakers will be encouraged to contact study investigators in case they have any questions or concerns regarding the study procedure.

### Data management {19}

Data will be managed by using REDCap® tools hosted by the University of Bergen [30]. REDCap is a web-based software solution that permits secure storage, analysis, and sharing of data by providing functions such as (i) user authentication and role-based security, (ii) intuitive electronic CRFs, (iii) real-time data validation and integrity checks, (iv) trails for tracking data manipulation and export procedures, and (v) export procedures for seamless data downloads to common statistical packages.

Tablet computers with REDCap software will be provided to research assistants and investigators. Passwords will be created for each research assistant and investigator to get access to the study file in REDCap. Research assistants will have access only to fill in the respective CRFs of the study participants they recruit at each visit, and they will have no access to change any information entered in REDCap. Only study investigators will have access to change information filled in REDCap when the need arises. All data with identifiers will be stored on firewall-protected secure servers, separately from clinical and microbiological data.

For enrolled infants, four different CRFs will be used for the study, three for the study visits and one for adverse events/unscheduled visits due to hospitalization/outpatient attendance for participants who fall sick during the study period. Infant deaths, hospitalizations, and sepsis episodes will also be recorded.

### Confidentiality {27}

Data with identifying variables will be stored on firewall-protected secure servers, separately from non-identifiable information such as clinical and microbiological data. Consent forms from mothers/caretakers will be safely stored separately from the collected data in a locked cabinet.

### Plans for collection, laboratory evaluation, and storage of biological specimens for genetic analysis in this trial/future use {33}

Standard operating procedures (SOP) which have been developed for the study will be used for specimen collection and processing. One fecal sample and one rectal swab sample will be collected at 6 weeks and 6 months visits, respectively. The fecal samples will be obtained using commercially available sampling kits (OMNIgen GUT kit, DNA Genotek, Ottawa, Canada) allowing storage of samples at ambient temperatures for up to 7–14 days before DNA extraction or freezing. These samples will be transported to HLH latest within 3 days after sampling and at HLH stored in − 80 °C freezers. The rectal swab samples will be taken by a research assistant using eSwab (Copan Diagnostics, CA, USA), transported to the laboratory at HLH the same day, and frozen at − 80 °C freezers until analysis.

## Statistical methods

### Statistical methods for primary and secondary outcomes {20a}

#### Clinical data

In univariate analysis, comparison of proportions will be done using Pearson’s chi-squared test and comparison of means will be done using *t* test or non-parametric tests as appropriate. Logistic regression will be used for multivariate analysis when assessing the relative importance of risk factors for the carriage of ESBL-E and other multi-resistant bacteria. The analysis will be performed in Stata 13 (Stata Corporation, College Station, Texas).

### Stool microbiota, stool metabolome, and stool inflammatory markers

#### Stool microbiota

A Poisson generalized linear model will be used to calculate trends in the relative abundance of genera and antibiotic resistance genes in the stool microbiota. Corrections based on multiple comparisons will be performed by the Benjamini-Hochberg false discovery rate (FDR). An FDR *Q* value ≤.10 will be considered significant for any analyses with multiple comparisons. A standard *P* value ≤.05 will be considered significant for all other analyses. Alpha diversity of the gut microbiota will be assessed by calculating the Shannon Diversity Index (MEGAN, v5.10.6). Multiple beta diversity metrics of samples will be performed by using non-metrical multidimensional scaling (NMDS), based on a matrix of Bray-Curtis distances and calculated by using the vegan R package. Differences between groups will be tested by using permutational multivariate analysis on beta diversity matrices.

Stool metabolites will be identified by using mass spectral databases (e.g., MzCloud, Metlin) and standards purchased for this project. For statistical and pathway analyses, we will use web-based tool MetaboAnalyst. All MS data will be submitted to and stored at the European Bioinformatics Institute repository MetaboLights.

Stool inflammatory markers will be analyzed with conventional statistical tools for interval data using parametric or non-parametric tests, as appropriate.

### Composition of the data monitoring committee, its role, and reporting structure {21a}

A data safety monitoring body (DSMB) has been appointed, which will periodically conduct scheduled, and in the case of AE, unscheduled reviews. The DSMB consists of our experts from both Norway and Tanzania. The DSMB will evaluate the accumulated study data with the main goal of ensuring that the rights and well-being of participants are protected. Site monitoring will also ensure that the conduct of the RCT adheres with the study protocol, good clinical practice (GCP), good laboratory practice (GCLP), and applicable regulatory requirements. In addition to ensuring the well-being of study participants, the DSMB will verify that the reported clinical and laboratory data are accurate, complete, and verifiable from the source data, ultimately providing recommendations to the ProRIDE study consortium concerning the continuation, modification, or termination of the trial, if necessary. The DSMB will always maintain confidentiality during all phases of DSMB review, including internal discussions and activities, and contents of reports provided by it. Usually, only voting members of the DSMB will have access to interim analyses of outcome data by treatment group. Exceptions may be made when the DSMB deems it appropriate. The DSMB is independent from the sponsor and has no competing interests.

### Interim analyses {21b}

We plan for interim analyses after the recruitment of 500 and 1000 infants. The major outcomes of interest; mortality, hospitalizations, and blood culture-confirmed sepsis will be summarized and presented to the DSMB. Only the study statistician and the PIs of the study, in addition to the DSMB, will have access to these results. If mortality between groups (probiotic vs placebo) is significantly different at assessment at these time points of the study, the study will be stopped. This decision will be taken by the PIs of the study, in consultation with co-investigators and the DSMB. Other triggers that would call for an unscheduled DSMB review are if infant mortality or morbidity rates clearly exceed expected rates in Tanzania.

### Methods for additional analyses (e.g., subgroup analyses) {20b}

Depending on the final results, it may be relevant to conduct subgroup analyses. Studies have shown that even short hospitalizations increase the risk of ESBL-E gut colonization [31, 32], and thus, it may be relevant to conduct subgroup analyses where participants will be stratified according to the place of birth (hospital/home).

### Methods in analysis to handle protocol non-adherence and any statistical methods to handle missing data {20c}

We will handle patient data according to the intention-to-treat principle. Patients will be compared—in terms of their final results—within the groups to which they were initially randomized, independently of receiving the allocated treatment, having dropped out of the study, or having violated the initial protocol (for whatever reason). This is a RCT and missing data will most likely occur at random between the two groups. We aim specifically to record a simple core set of clinical primary outcomes (mortality and morbidity assessed primarily as hospitalization) in order to minimize the occurrence of missing values. Missing data will be discarded from further analysis, and thus, participants with missing data on the primary outcome will be omitted from the main analysis.

### Plans to give access to the full protocol, participant level-data, and statistical code {31c}

#### Data sharing statement

The raw data from this study will be made available by the authors, without undue reservation, to any qualified researcher no later than 3 years after the publication of the main study. Moreover, we will deliver a completely de-identified data set to an appropriate data archive for sharing purposes. Pathogen genomes that are sequenced will be made available for other researchers in GenBank®.

## Oversight and monitoring

### Composition of the coordinating center and trial steering committee {5d}

The consortium consists of partners that have competence in diverse and complementary scientific fields, including infectious disease, pediatrics, clinical microbiology, and bioinformatics. NL, who will lead the consortium, BB, and SJM have experience in clinical studies in low-income settings with a particular focus on antimicrobial resistance. All of these partners (NL, BB, SJM) have also conducted research regarding ESBL-E resistance in children. CK is an experienced pediatrician/neonatologist with experience from clinical work in Tanzania, as well as research on infections and the use of antibiotics in neonates. He has recently completed a study using metagenome analyses of gut microbiota and resistome in stool samples of preterm infants. Clinical microbiologists IL and JM have experience in research on ESBL-E and will be in charge of overseeing laboratory procedures in Tanzania and Norway. JG at the Pediatric Department is the HLH partner and local clinical principal investigator.

### Adverse event reporting and harms {22}

The DSMB are in charge of conducting unscheduled reviews in the case of adverse events, ultimately providing recommendations to the ProRIDE study consortium concerning the continuation, modification, or termination of the trial.

### Frequency and plans for auditing trial conduct {23}

Site monitoring will be conducted to ensure that the rights and well-being of study participants are protected. In addition, monitoring will verify that the reported clinical and laboratory data are accurate, complete, and verifiable from source data. Site monitoring will also ensure that the conduct of the clinical trial is in adherence with the study protocol, good clinical practice (GCP), and good laboratory practice (GCLP) and applicable regulatory requirements (Tables 2 and 3).

### Plans for communicating important protocol amendments to relevant parties (e.g., trial participants, ethical committees) {25}

Any recommendations from the DSMB will firstly be communicated to the consortium. If changes in protocol are to be made, the following relevant parties will be informed:

- National Institute of Medical Research in Tanzania (NIMR), Tanzania
- Tanzania Medicines and Medical Devices Authority (TMDA), Tanzania
- The Regional Committee for Medical and Health Research Ethics Western Norway, Norway

### Dissemination plans {31a}

The final report will be prepared by the Principal Investigator and Co-investigators. Study participants will be informed of the study results. The health care workers at HLH where the study will be conducted will be informed of the results obtained. The results will also be made available to the Ministry of Health and Social Welfare. Study findings will also be published in international peer-reviewed journals, preferably in journals which are available free of charge, so that the information can be accessible to health professionals in the settings where the study will be conducted. In addition, results will be given to participating hospitals and to governmental bodies responsible for guidelines for infant treatment and prophylaxis.

## Discussion

Despite promising results regarding the use of probiotics to reduce the carriage of MDR bacteria, no RCT has investigated whether probiotics may reduce ESBL-E gut colonization in infants [16, 25]. Therefore, the ProRIDE Trials aim to evaluate whether the use of probiotics can reduce infection-related morbidity and mortality by preventing ESBL-E colonization and/or infections among infants in Haydom, Tanzania. The RCT is a large-size study with a rigorous design. By using a large sample size and applying bias-reducing techniques, such as randomization and blinding, we can provide robust evidence that is necessary to evaluate whether probiotics can significantly reduce morbidity and mortality related to ESBL-E colonization among infants in Haydom, Tanzania.

### Methodological considerations

Our aim is to recruit 2000 infants in Haydom and the surrounding area, more specifically newborns who are delivered at HLH or at home. Recruiting newborns delivered at HLH is expected to be feasible due to experienced research infrastructure and available research nurses at HLH. We expect more challenges in identifying and recruiting women who plan to deliver at home. Own fieldworkers will be in charge of conducting field visits to identify potential mothers in their last trimester, screening newborns, and enrolment and follow-up of eligible newborns. Although this may be challenging, HLH has long-standing experience with running mobile clinics and conducting community research [33]. Furthermore, the recruitment of newborns delivered outside the hospital is of importance due to two reasons. Firstly, women who give birth at home may be significantly different from women delivering at the hospital in terms of socioeconomic status [34, 35]. Utilization of health care services and delivery at the hospital is costly due to traveling and consultation fees, and women who cannot afford these expenses may choose to deliver at home. Given that ESBL-E gut colonization is associated with lower socioeconomic status [36, 37], it is important that infants of such families are recruited in order to obtain a representable sample of the population of interest. Secondly, because hospitalization is associated with ESBL-E colonization, exclusively recruiting newborns delivered at the hospital may yield a sample where the baseline colonization rate does not truly reflect the baseline in the population of interest.

Probiotics/placebo drops will be administered at home by mothers, and thus, we cannot directly observe if the infants are receiving the drops appropriately. However, during the recruitment of mothers in their last trimester, enrolment of newborns, and the first follow-up visit, mothers will be explained how the drops are to be administered and the importance of completing the course appropriately. Moreover, adherence will also be evaluated during the first and second follow-up visits by observing the content of the bottles.

### Ethical equipoise and risk

There is a genuine uncertainty about the effect of the intervention on morbidity and mortality compared to what is being offered to the control group—placebo. The Panigrahi trial was performed in a different continent with a probiotic product combined with a prebiotic [15]. The effects observed in India were strikingly positive, but need to be confirmed in other large trials before this can be introduced as standard care for newborn infants in low-income countries.

There is currently no recommended therapy for preventing ESBL-E gut colonization and conducting this trial does not breach the ethical equipoise. Furthermore, included infants—both in the intervention arm and control arm—who may be sick and need hospitalization for any illnesses will receive standard care treatment at HLH, including study-funded optimal assessment of a potential infection with blood culture and CRP analyses (ancillary care).

Infants included in this trial will not undergo any painful procedures, in particular, no extra blood samples will be obtained. The IP is not likely to cause severe side effects or harm [19], given the well-known safety of probiotics in infants, and the lack of known interaction with other important drugs used for any other required therapy. Therefore, it is considered that the risk associated with trial participation is minimal. Even though the study products are not considered to be medicinal drugs, and studies have so far not revealed any serious adverse effects, study participants will also be insured, if unforeseen side effects of the probiotics develop.

### Local support

HLH has a strong and well-established research infrastructure. It has recently been involved in larger studies such as MAL-ED [33] and Helping Babies Breathe [38], and over the past 10 years, the hospital has been involved in community research, which has built trust between the hospital and the community around [39]. To explore the attitude and acceptability towards the intervention, we will also, before study-start, perform focus group discussions with women caring for infants. Advice from the mothers in the ProRIDE Trial will be particularly valuable if the trial results are to be implemented in routine infant care.

### Potential impact

The hypothesis in the ProRIDE Trial is that probiotics will significantly reduce hospitalization and death (as a composite outcome) up to 6 months of age. Moreover, we hypothesize that the intervention may be associated with a reduction in ESBL-E-carriage and infections. Thus, the ProRIDE trial will benefit participants, caretakers, and health personnel, with the aim of ensuring improved clinical health of participants and obtaining an in-depth understanding of the effects of probiotics in children. It is a simple intervention that is realistic to implement even outside a controlled, research environment. Because probiotics does not need to be administered in the hospital or by a health professional, and is to our knowledge not associated with any serious adverse side effects, it is likely that it can be administered at home by caretakers.

If the hypothesis of the ProRIDE Trial is confirmed, the study results will also be helpful for government officials responsible for national guidelines, as it will support the implementation of probiotic use in routine clinical care to reduce morbidity and improve survival of infants in low-income settings. If the study confirms probiotics as an efficient tool to reduce carriage of ESBL-E, this may also have implications far beyond the study setting, including in Norway, as ESBL-E carriage has rapidly developed into a global challenge. Lastly, AMR is posing a major threat to human health globally, and a reduction in the prevalence of AMR would subsequently lead to reduced consumption of broad-spectrum antibiotics and thereby reduce drivers of resistance.

### Trial status

At the time of this submission, the trial has been approved by REK, NIMR, and TMDA. Recruitment of participants was expected to start in November 2020 and be completed in November 2021. However, the current COVID-19 pandemic has already substantially delayed the study start, and there is also an uncertainty around further development of the pandemic and its impact on clinical research in Tanzania, and many other countries in the world. We now are planning to start the study in the second half of 2021.

Protocol version: March 2021, version 1.1.

## Supplementary Information

**Additional file 1.** 7221 Probiotika til nyfødte for å redusere kolonisering og sykdom med resistente bakterier.

**Additional file 2.** Letter of ethical review REC West.

**Additional file 3.** Re: ethical clearance certificate for conducting medical research in Tanzania.

**Additional file 4.** Tildeling av Helse Vests forskingsmidlar 2019 - Åpen prosjektstøtte.

**Additional file 5.** Letter of financial support.

## Abbreviations

AE/SAE: Adverse events/serious adverse events
AMR: Antimicrobial resistance
BSI: Bloodstream infection
CRF: Case report form
DSMB: Data safety monitoring body
DTA: Data transfer agreement
ESBL: Extended-spectrum beta-lactamase Enterobacteriaceae
ESBL-E: Extended-spectrum beta-lactamase-producing
eCRF: Electronic case report forms
FDR: False discovery rate
GCP: Good Clinical Practice
GCLP: Good Laboratory Practice
GMP: Good Manufacturing Processes
GPP: Good Production Practices
HLH: Haydom Lutheran Hospital
ICF: Informed consent form
IP: Investigational product
IRB: Ethical approval
MDR: Multi-drug-resistant
MTA: Material transfer agreement
NIMR: National Institute of Medical Research in Tanzania
NMDS: Non-metrical multidimensional scaling
RCT: Randomized controlled trial
REK: Regional Committee for Medical and Health Research Ethics of Western Norway
WHO: World Health Organization
