# Supplementary material for: Use of probiotics to reduce infections and death and prevent colonization with extended-spectrum beta-lactamase (ESBL)-producing bacteria among newborn infants in Tanzania (ProRIDE Trial): study protocol for a randomized controlled clinical trial
Source: Trials. 2021 Apr 29;22:312. doi: 10.1186/s13063-021-05251-3 (PMC8082054; doi:10.1186/s13063-021-05251-3)
Supplement: Supplementary file 1 — Additional file 1. 7221 Probiotika til nyfødte for å redusere kolonisering og sykdom med resistente bakterier. [file 13063_2021_5251_MOESM1_ESM.pdf]

Region:	Saksbehandler:	Telefon:	Vår dato:	Vår referanse:
REK vest	Fredrik Kolstad Rongved		04.09.2019	7221
				Deres referanse:

Nina Langeland

7221 Probiotika til nyfødte for å redusere koloniering og sykdom med resistente bakterier

Forskningsansvarlig: Universitetet i Bergen

Prosjektleder: Nina Langeland

Prosjektleders prosjektomtale

Prosjektet er en dobbelt-blindet randomisert kontrollert studie hvor nyfødte barn født til termin i Haydom, Tanzania, får enten dråper med probiotika eller placebo-dråper daglig i en måned. I vestlige land er det vist at probiotika gitt til premature barn bedrer prognosen deres. I India er det gjennomført en studie av barn født til termin der det ble vist at symbiotika (lignende men ikke det samme som probiotika) reduserte sykehusinnleggelser hos barn under ett år, etter behandling med symbiotika i en uke. Vår hypotese er at probiotika reduserer kolonisering med resistente bakterier i tarmen hos nyfødte og at dette er mekanismen for redusert sykelighet. Primærendepunkt i studien er kombinert utkomme av død og/eller sykehusinnleggelser i 6 måneder etter fødsel, sekundærendepunkt er bærerskap i tarm av resistente bakterier, barnets vekst, sepsis-episoder og mikrobiota-sammensetningen i tarmen ved 6 uker og 6 måneders alder. Sykehusinnleggelser i inklusjonsperioden danner en substudie.

REKs vurdering

Vi viser til søknad om forhåndsgodkjenning av ovennevnte forskningsprosjekt innsendt 11.06.2019. Søknaden ble behandlet av Regional komité for medisinsk og helsefaglig forskningsetikk (REK vest) i møtet 14.08.2019. Vurderingen er gjort med hjemmel i helseforskningsloven (hforsknl) § 10

Forsvarlighet:

Kontrollgruppen får standardbehandling. Det skal tas blodprøver og avføringsprøver. Det blir gitt behandling for andre sykdommer som malaria. Primærendepunkt i studien er kombinert utkomme av død/eller sykehusinnleggelser i 6 måneder etter fødsel. Barnet skal få probiotika daglig de første fire ukene:

«Five drops (0.2 ml) contain 1.8 x 109 CFU Lactobacillus acidophilus, Bifidobacterium infantis and Bifidobacterium breve (equal amount of all three strains) will be given by mouth once daily for 4 weeks to "bottle empty". Bottle contains 5-5.5 ml.» (Protokoll s. 21)

Prosjektleder selv påpeker at det kan være ugunstig for barna som får placebo dersom det blir påvist nytte av probiotika. Prosjektgruppen vil derfor gjøre en interimanalyse når halvparten av barna er inkludert, med tanke på forskjeller i dødelighet. Det er etablert en Data Safety Monitoring Committee (DSMC), og lokal PI har tatt GCP-kurs. Prosjektmedarbeidere i Tanzania vil være tilgjengelige for mødrene per telefon.

Komiteen vurderer at dette er en interessant og viktig studie med liten risiko. Prosjektet oppfyller lovens krav for forskning på mennesker uten samtykkekompetanse, jf. helseforskningsloven § 18. Komiteen anser prosjektet som forsvarlig å gjennomføre under forutsetning at prosjektet blir vurdert av, eller har et samarbeid med, en lokal pediater. Komiteen legger også til grunn at prosjektet får de lokale tillatelser av Tanzanian Food and Drug Authority dersom det kreves etter lokal lov.

Barn innlagt på sykehus med feber (delprosjekt):

I tillegg til den randomiserte studien, vil dette prosjektet samle inn opplysninger fra alle barn under ett år som kommer til sykehuset med feber. Det er lagt ved et eget samtykkeskjema for dette formål. De inkluderte barna i denne separate studien vil være både deltakere i den randomiserte hovedstudien samt andre barn som ikke deltar.

Disse barna vil avgi 4-5 ml blod for å lage blodkultur hvor en kan sjekke for infeksjoner. I tillegg vil det bli tatt en *rectal swab* prøve for å undersøke korrelasjon mellom *«… gut carriage of ESBL-E [extended spectrum beta-lactamase producing Enterobacteriales] and ESBL-E as a cause for BSI [Blood Stream Infection]»*. (Protokoll s. 25)

Komiteen forutsetter at det gjøres en konkret vurdering av blodvolum som det enkelte barn kan avgi ved infeksjon.

Helseopplysninger:

Opplysninger fra svangerskapsjournal hos mor og sykejournal hos barna ved innleggelser. I tillegg registreres data fra kontroller ved 6 uker og 6 måneder. Komiteen har ingen merknader.

Humant biologisk materiale:

Det er ønskelig å ta blod- og avføringsprøver fra barna, i tillegg til bakterieisolater. Materialet skal lagres i en tidligere godkjent generell forskningsbiobank kalt *«Diagnostisk og forskningsbiobank for primære/ervervede immunsvikttilstander og alvorlige infeksjonssykdommer»*, ansvarshavende Nina Langeland (REK vest ref. 165.04). Komiteen har ingen merknader.

Rekruttering:

Det vil bli inkludert 1000 barn i probiotika-gruppen og 1000 i kontrollgruppen. Bare friske nyfødte uten synlige misdannelser eller påvist annen sykdom vil bli inkludert. Vekt ved fødsel ≥ 2 kg. Mødrene kontaktes ved siste svangerskapskontroll på sykehuset, eller ved hjemmebesøk i siste trimester. For delprosjektet vil rekruttering skje ved innleggelse. Komiteen har ingen merknader.

Informasjonsskriv og samtykkeskjema:

REK vest ber om at informasjonsskrivet revideres i henhold til følgende merknader:

- Forskningsansvarliges logo må fremkomme på informasjonsskrivet.
- Informasjonsskrivet må informere om at det er en randomisert kontrollert studie hvor halvparten får placebo. I skrivet virker det som at alle skal få probiotika, hvilket ikke er tilfelle.
- Det må opplyses om risikoen ved prosjektet.

Revidert informasjonsskriv skal sendes til REK vest gjennom «oppgaver» i den nye portalen.

Innføring av data og prøver:

Data og prøver skal sendes til Norge. Av materiale skal avføringsprøver og bakterieisolater sendes til Norge for analyse og oppbevaring. Restmateriale etter analyse vil lagres i en biobank i Norge. Dataoverføringsavtale (DTA) og materialoverføringsavtale (MTA) vil bli signert mellom Haydom og UiB. Det overføres ikke personidentifiserbare data til Norge. Komiteen har ingen merknader.

Prosjektslutt og behandling av data:

Prosjektslutt er satt til 01.10.2024. Ved prosjektslutt skal data anonymiseres. Komiteen har ingen merknader.

Vedtak

Godkjent med vilkår

REK vest setter følgende vilkår:

- Prosjektet må legges fram for vurdering av en lokal pediater.
- Lokale myndigheter må vurdere om prosjektet skal framlegges for lokalt legemiddelverk.
- Revidert informasjonsskriv skal sendes til REK vest.

Vedtakstekst*:* REK vest har gjort en helhetlig forskningsetisk vurdering av alle prosjektets sider. Prosjektet godkjennes med hjemmel i helseforskningsloven § 10 på betingelse av at ovennevnte vilkår tas til følge.

Med vennlig hilsen
Marit Grønning
Prof. dr.med.
Komiteleder

Fredrik Rongved
rådgiver
