# Supplementary material for: Use of probiotics to reduce infections and death and prevent colonization with extended-spectrum beta-lactamase (ESBL)-producing bacteria among newborn infants in Tanzania (ProRIDE Trial): study protocol for a randomized controlled clinical trial
Source: Trials. 2021 Apr 29;22:312. doi: 10.1186/s13063-021-05251-3 (PMC8082054; doi:10.1186/s13063-021-05251-3)
Supplement: Supplementary file 2 — Additional file 2. Letter of ethical review REC West. [file 13063_2021_5251_MOESM2_ESM.pdf]

REK
REGIONALE KOMITEER FOR MEDISINSK OG HELSEFAGLIG FORSKNINGSETIKK

Regional Committee for Medical and Health Research Ethics, Western-Norway

To whom it may concern

Our ref	Date
2019/1025	21.11.2019

Confirmation

I hereby confirm that the project *"Use of Probiotics to Reduce Infections and Death and Prevent Colonization with Extended-spectrum beta-lactamase (ESBL) producing bacteria, among newborn infants in Haydom and surrounding area, Tanzania, a randomized controlled clinical trial"*, by project manager Nina Langeland, is reviewed and approved by the Regional Committee for Medical and Health Research Ethics, Western-Norway.

Best regards

Fredrik K. Rongved

Fredrik Rongved
Committee secretary

Postadresse
Postboks 7804
5020 Bergen

rek-vest@uib.no
helseforskning.etikkom.no/xnet/public
Org no. 874 789 542

Regional komité for medisinsk forskningsetikk, Vest-Norge
Telefon 55 97 84 97 / 98 / 99

Besøksadresse
Haukeland Universitetssykehus
