# Supplementary material for: Use of probiotics to reduce infections and death and prevent colonization with extended-spectrum beta-lactamase (ESBL)-producing bacteria among newborn infants in Tanzania (ProRIDE Trial): study protocol for a randomized controlled clinical trial
Source: Trials. 2021 Apr 29;22:312. doi: 10.1186/s13063-021-05251-3 (PMC8082054; doi:10.1186/s13063-021-05251-3)
Supplement: Supplementary file 3 — Additional file 3. Re: ethical clearance certificate for conducting medical research in Tanzania. [file 13063_2021_5251_MOESM3_ESM.pdf]

THE UNITED REPUBLIC OF TANZANIA

National Institute for Medical Research
3 Barack Obama Drive
P.O. Box 9653
11101 Dar es Salaam
Tel: 255 22 2121400
Fax: 255 22 2121360
E-mail: nimrethics@gmail.com

Ministry of Health, Community
Development, Gender, Elderly & Children
University of Dodoma, College of
Business Studies and Law
Building No. 11
P.O. Box 743
40478 Dodoma

NIMR/HQ/R.8a/Vol. IX/3398

9th April, 2020

Dr. Joshua Gidabadya
Paediatrician
Haydom Lutheran Hospital
P. O. Box 9000
Manyara

RE: ETHICAL CLEARANCE CERTIFICATE FOR CONDUCTING MEDICAL RESEARCH IN TANZANIA

This is to certify that the research entitled: Use of Probiotics to Reduce Infections and Death and Prevent Colonization with Extended-spectrum beta-lactamase (ESBL) producing bacteria, among newborn infants in Haydom and surrounding area, Tanzania, a randomized controlled clinical trial (Gidabadya J. et al), has been granted ethical clearance to be conducted in Tanzania.

The Principal Investigator of the study must ensure that the following conditions are fulfilled:

1. Progress report is submitted to the Ministry of Health, Community Development, Gender, Elderly & Children and the National Institute for Medical Research, Regional and District Medical Officers after every six months.
2. Permission to publish the results is obtained from National Institute for Medical Research.
3. Copies of final publications are made available to the Ministry of Health, Community Development, Gender, Elderly & Children and the National Institute for Medical Research.
4. Any researcher, who contravenes or fails to comply with these conditions, shall be guilty of an offence and shall be liable on conviction to a fine as per NIMR Act No. 23 of 1979, PART III Section 10(2).
5. Sites: Haydom Lutheran Hospital.

Approval is valid for one year: 9th April, 2020 to 8th April, 2021.

Name: Prof. Yunus Daud Mgaya

Signature
CHAIRPERSON
MEDICAL RESEARCH
COORDINATING COMMITTEE

Name: Prof. Muhammad Bakari Kambi

Signature
CHIEF MEDICAL OFFICER
MINISTRY OF HEALTH, COMMUNITY DEVELOPMENT, GENDER, ELDERLY & CHILDREN

CC: Director, Health Services-TAMISEMI, Dodoma
RMO of Manyara region.
DMO/DED of Mbulu district.
