# Supplementary material for: Use of probiotics to reduce infections and death and prevent colonization with extended-spectrum beta-lactamase (ESBL)-producing bacteria among newborn infants in Tanzania (ProRIDE Trial): study protocol for a randomized controlled clinical trial
Source: Trials. 2021 Apr 29;22:312. doi: 10.1186/s13063-021-05251-3 (PMC8082054; doi:10.1186/s13063-021-05251-3)
Supplement: Supplementary file 4 — Additional file 4. Tildeling av Helse Vests forskingsmidlar 2019 - Åpen prosjektstøtte. [file 13063_2021_5251_MOESM4_ESM.pdf]

Regionalt samarbeidsorgan for forsking og innovasjon | HELSE VEST | Høgskulen på Vestlandet | Universitetet i Stavanger | UNIVERSITETET I BERGEN

Helse Bergen HF
Medisinsk avdeling
v/Nina Langeland

Dykkar ref.:	Vår ref.: **912267**	Bergen 19. desember 2018

Tildeling av Helse Vests forskingsmidlar 2019 - Åpen prosjektstøtte

På møtet i Det regionale samarbeidsorganet for forsking og innovasjon 28. november 2018, vart Helse Vests søkbare forskingsmidlar for 2019 fordelt.

Forskingsprosjektet *« Probiotics to prevent ESBL colonization among newborn infants in Tanzania»*, har blitt tildelt kr. **1 200 000** for 2019 og fått Helse Vest prosjektnummer**: 912267.** Midlane er løyvde berre til dette prosjektet og skal nyttast i tråd med beskrivinga i søknaden. Ansvarleg institusjon er Helse Bergen HF.

Kreditering: Vi minner om at eit helseføretak i regionen skal krediteras i ein publikasjon dersom den har gitt eit nødvendig og vesentleg bidrag til en forfattars medverknad til det publiserte arbeidet, sjå *kreditering av forskingsproduksjon* .

Tal år: Forutsatt framdrift som skal dokumenterast gjennom fagleg rapportering og rekneskapsrapportering, vil prosjektet tildeles midlar også i 2020 og 2021, høvesvis kr. 1 300 000 og 1 600 000.

Merk!

Stillingar finansiert av Helse Vests forskingsmidlar skal tilsetjast i eit helseføretak, her ved Helse Bergen HF. Institusjonen fastsetter løn innanfor dei rammer som gjeld i helseføretaket.

Andre forhold

Du har opplyst om at søknad til REK om godkjenning av prosjektet er eller vil bli sendt. Vi gjer merksam på at prosjektleiar er ansvarleg for at gjeldande lover og forskrifter rundt forskingsprosjekta er avklart før datainnsamling startar, jf også tildelingsbrevets vedlegg. Ved aksept av midlane godtek du samtidig vilkåra for Helse Vests forskingsmidlar som er skildra under.

Ekstern finansiering
Samarbeidsorganet legg til grunn at det blir søkt om finansiering frå EU-systemet ved neste aktuelle utlysing. Prosjektansvarlege vil bli bedne om å rapportere på dette i samband med den årlege faglege rapporteringa

Med venleg helsing
Regionalt samarbeidsorgan for forsking og innovasjon

Sølvi Lerfald
Fagleiar

E-kopi: Helse Vest v/Marte Alise Ørke Larsen
Avdelingsdirektør Solveig Hansen
Rekneskapsførar Therese Espeland Mowatt

Helse Vest forskingsmidlar

Det regionale samarbeidsorganet mellom Helse Vest og universitet- og høgskulesektoren i regionen får sitt mandat frå styret i Helse Vest, og skal blant anna fatte vedtak i saker om fordeling av det statlege øyremerka tilskotet til forsking. Desse midlane skal være eit insentiv og økonomisk bidrag til å sikre forsking og oppbygging av forskingskompetanse i helseføretaka, jf. Statsbudsjettets omtale av midlane (kap.732, post 78).

I søknaden om forskingsmidlar skal det være ein plan for faktiske kostnader og dekning av desse. Dette skal være avklart på førehand, og nedfelt i avtaler med eventuelle eksterne partar. Når tildelinga aksepterast av søkjaren, godtek søkjar samtidig at prosjektet kan gjennomførast med tilgjengelege ressursar.

Endringar i prosjektperioden

- Søknad om foreldre- og omsorgspermisjon sendast til arbeidsgivar, dvs. den institusjonen der du har inngått arbeidskontrakt. Prosjektansvarleg skal melde frå om permisjonen (frå dato - til dato) til samarbeidsorganet sitt sekretariat, (forskningregnskap@helse-vest.no) så snart som mogleg. Sjå for øvrig retningslinjer for permisjonar: https://helse-vest.no/vart-oppdrag/vare-hovudoppgaver/forsking/forskingsmidlar/retningslinjer-og-skjema/retningslinjer-for-permisjonar

- Eventuelle andre forseinkingar og endringar skal også meldast inn til samarbeidsorganets sekretariat så snart som mogleg. Dersom dette fører til endringar i tildelingsperioden, skal det sendast søknad med grunngjeving.

Handtering og utbetaling av midlane

- Helse Vests forskingsmidlar skal handteras av et helseføretak i regionen eller ein privat ideell institusjon som har avtale med Helse Vest. Sjå retningslinjer for handtering av forskingsmidlane.
- Rekneskapsførande institusjon skal sende samlefaktura til **Helse Vest RHF, Lønns- og regnskapssenteret, Postboks 314, 4066 Stavanger** månadleg basert på forbruk føregåande månad. Ved fleire prosjekt oversendast vedlegg som viser forbruk per prosjekt.

- Ved anskaffing av utstyr for forskingsmidlane må det førelegga godkjenning frå føretaket som skal være eigar av utstyret. Midlane skal berre nyttast til formål det er søkt om.

Generelle rutinar for regnskaps- og faglig rapportering

Rapportering av aktivitet og rekneskap er obligatorisk. Manglande rapportering vil ha konsekvensar for eventuell seinare tildeling.

- **Fagleg rapport** skal leverast **rundt 20. januar**. Eksakt frist vert oppgitt ved utsending av rapportskjema. Rapporteringa skjer elektronisk og rapportskjema sendast til den som har fått midlane. For at rapportering skal skje på korrekt måte, er det viktig at riktige e-postadresser meldast til forskningsregnskap@helse-vest.no.
- **Årsrekneskapet** skal leverast i **januar**. Også rekneskapsrapporteringa skjer elektronisk. Rekneskapsskjema sendast til den som er oppgitt som rekneskapsfører, her er det viktig at riktig e-postadresse til rekneskapsførar meldast til forskningsregnskap@helse-vest.no.
- Dersom det vert aktuelt å **overføre midlar** til neste kalenderår, må retningslinjene for overføring av midlar følgjast. Det er mogleg å søkje overføring av ubrukte midlar i forbindelse med rekneskapsrapporteringa med **frist i januar**. Dette inneber at det er feltar i det elektroniske rekneskapsskjemaet kor det kan søkjast om overføring av midlar og kor det skal grunngjevast kvifor man trenger overføring. Dersom ubrukte midlar ikkje søkjast overført, vert midlane trekt tilbake til Samarbeidsorganet.
- Helse Vests forskingsmidlar skal sikre at det vert forska i helseføretaka. Helse Vests revisor har til ein kvar tid rett til å kontrollere at midlane vert forvalta i samsvar med vilkåra i tildelinga. Vi gjer merksame på at du pliktar å gi Helse Vest RHF sin revisor dei opplysningane som dei krev for sin kontroll. Riksrevisjonen har også anledning til slik kontroll, jf. Stortingets bevilgningsreglement § 10.
- Helse- og omsorgsdepartementet har fastsatt at det berre er helseføretaka og dei private, ideelle institusjonane[1] som har avtale med Helse Vest, som kan være rekneskapsførande institusjon for tildelingar av nye forskingsprosjekter frå Helse Vest.

- Vi føreset at den enkelte gjer seg kjent med dei retningslinjer og rutinar for forskingsrekneskap som fins ved eigen arbeidsstad.

- Vi føreset også at den enkelte gjer seg kjent med dei retningslinjer og rutinar for innovasjon som fins ved det helseføretak der søknaden er forankra. Retningslinjer for handtering av innovasjon finn du på helseføretaka sine nettsider.

Kreditering av vitskapelege artiklar

Formålet med Helse Vests forskingsmidlar er at dei skal medverke til å sikre forskingsaktivitet i helseføretaka, og auke produktiviteten og kvaliteten på forskinga. Forskingsaktiviteten målast av Helse- og omsorgsdepartementet årleg og får direkte konsekvensar for kor mykje som kan tildelast av forskingsmidlar dei tre påfølgjande år.

I samsvar til brev frå Helse- og omsorgsdepartementet (HOD) den 25. mars 2008 så bør dei statlege øyremerka midlane som vert fordelt av Helse Vest og det regionale samarbeidsorganet:

> *«tildeles institusjoner og enheter som inngår i det nasjonale målesystemet for forskningsresultater i helseforetakene. Dette dokumenteres gjennom kreditering til den samme institusjonen ved å angi en korrekt adresse på publikasjonene. Det må være en entydig adresse til et av helseforetakene eller institusjonene som er tilknyttet det*

nasjonale målesystemet. På samme måte vil det gjelde rapportering av gjennomførte doktorgradsarbeid i henhold til kriteriene i det nasjonale målesystemet for forskningsresultater.»[MVT1]

Forskingsaktiviteten målast i tal doktorgrader utført ved eller finansiert av eit helseføretak samt vitskapelege artiklar med ein forfattaradresse som viser til eit sjukehus i regionen. Merk at forskarar som ikkje er tilsett ved eit sjukehus i regionen også kan oppgje ein forfattaradresse til sjukehuset kor forskinga er utført. Det fins eigne retningslinjer for korleis forfattarar kan kreditere institusjonar, sjå retningslinjer for kreditering av vitskaplege publikasjonar . Desse retningslinjene er i samsvar med fellesinstruksen frå Kunnskapsdepartementet og Helse- og omsorgsdepartementet.

Hovudregel

Det er den enkelte forskar som avgjer kreditering ut frå følgjande hovudreglar:

- Ein institusjon skal vere oppgitt som adresse i ein publikasjon dersom den har gitt eit naudsynt og vesentleg bidrag til eller grunnlag for ein forfattar sin medverknad til det publiserte arbeidet.
- Same forfattar skal også gje andre institusjonar sine adresser dersom desse i kvart einskild høve også fyller kravet i punkt 1.

Finansiering er eit av dei kriteriene som reknast som eit vesentlig bidrag i denne samanheng. Vancouver-reglene for forfattarskap kan tolkast analogt med kva for ein institusjon som kan krediteras i oppgitte forfattaradresser. Andre bidragsytarar kan førast opp under "acknowledgements".

Ved rapportering vil du verte spurd om å oppgje kva vitskapelege artiklar og doktorgrader som er eit resultat av forskingsmidlane tildelt av det Regionale samarbeidsorganet for forsking og innovasjon. Opplysningane du rapporterer inn vil verte samanstilt og kontrollert opp mot opplysningar registrert i CRIStin med Norsk vitskapsindeks. Alle forskarar kan logge på CRIStin for oversikt over egen forskingsaktivitet. Spørsmål om CRIStin kan rettes til cristin@helse-vest.no.

Eksemplar på adressering som gjer utteljing:

- Department of Surgery, Forde Hospital
- Department of Thoracic Medicine, Haukeland University Hospital
- Department of Pathology, Stavanger University Hospital

Eksemplar på tvitydig (feil) adressering:

- Institute for Internal Medicine, Haukeland University Hospital, University of Bergen
- Department of Thoracic Medicine, University of Bergen
- Department of Psychiatry, Stavanger University Hospital, University of Bergen

Dei overnemnde er tvitydig fordi det adresserast til to institusjonar på same linje. Her er det vanskeleg å sjå kva for ein institusjon det faktisk adresserast til. Viss det er riktig å adressere til to institusjonar skal ein dele opp adressene til dei to institusjonane på to linjer, eller to fotnotar.

Lover og forskrifter

Helseføretaket er ein forskingsansvarlig institusjon i samsvar til Lov om medisinsk og helsefaglег forsking[2] og har det overordna ansvaret for forskingsprosjekt og forskingsbiobanker. Prosjektleiar er ansvarleg for at gjeldande lover og forskrifter rundt forskingsprosjektet er avklart før datainnsamling startar. Ved den årlige faglige rapporteringa vil informasjon om dette verte etterspurd og kontrollert.

Medisinsk og helsefagleg forsking på folk, humant biologisk materiale (forskingsbiobank) eller helseopplysningar skal på førehandgodkjennast av den regionale etikkomiteen (REK)[3]. Alle føretaka har internkontrollsystem for forsking med rutinar frå oppstart til avslutning av forskingsprosjektar samt malar for informasjons- og samtykkeskjema mv. Prosjektleiarar pliktig til å følgje disse rutinane i gjennomføringa av forskingsprosjektet. Merk at UiB og Helse Bergen har utvikla eit felles internkontrollsystem, sjå http://forskning.ihelse.net.

Legemiddelutprøving skal godkjennast av Statens legemiddelverk (SLV)[4] og klinisk utprøving av medisinsk utstyr skal godkjennast av Helsedirektoratet[5]. Dersom det nyttast forsøksdyr i prosjektet føresetjast det at klarering føreligg frå Forsøksdyrsutvalet.

Forskingsprosjekt som ikkje er helseforsking skal godkjennast av Personvernombudet i det enkelte helseføretak. Prosjekt med eit særleg stort omfang føreset godkjenning frå Datatilsynet.

---

[1] http://www.helse-vest.no/no/Behandlingsstader/Sider/Private,-ideelle-institusjonar.aspx

[2] https://lovdata.no/dokument/SF/forskrift/2000-12-15-1265/KAPITTEL_7-4#§7-27

[3]https://helseforskning.etikkom.no/ikbViewer/page/reglerogrutiner/soknadsplikt?p_dim=34997&_ikbLanguageCode=n

[4] https://legemiddelverket.no/godkjenning/klinisk-utproving/soknad-om-klinisk-utproving#1

[5] https://helsedirektoratet.no/medisinsk-utstyr/klinisk-utproving-av-medisinsk-utstyr
