# Supplementary material for: Use of probiotics to reduce infections and death and prevent colonization with extended-spectrum beta-lactamase (ESBL)-producing bacteria among newborn infants in Tanzania (ProRIDE Trial): study protocol for a randomized controlled clinical trial
Source: Trials. 2021 Apr 29;22:312. doi: 10.1186/s13063-021-05251-3 (PMC8082054; doi:10.1186/s13063-021-05251-3)
Supplement: Supplementary file 5 — Additional file 5. Letter of financial support. [file 13063_2021_5251_MOESM5_ESM.pdf]

Regionalt samarbeidsorgan for forsking og innovasjon | HELSE VEST | Høgskulen på Vestlandet | Universitetet i Stavanger | UNIVERSITETET I BERGEN

To whom it may concern

Your ref.: Our ref.: F-10465/912267 Bergen 25.8.2020

**Letter of financial support**

We hereby confirm that Nina Langeland has received a grant for the research project "*Probiotics to prevent ESBL colonization among newborn infants in Tanzania*»**,** from the Western Norway Regional Health Authority. The total amount of the support is $457,420.68 USD (current date exchange rate) in the period 2019-2021.

Yours sincerely,
The Western Norway Regional Health Authority

Director of research
Haukeland University Hospital
Norway
